# From trials to communities: implementation and scale-up of health behaviour interventions

**DOI:** 10.1186/s12961-023-01027-0

**Published:** 2023-07-31

**Authors:** Sam McCrabb, Alix Hall, Heather McKay, Sharleen Gonzalez, Andrew Milat, Adrian Bauman, Rachel Sutherland, Luke Wolfenden

**Affiliations:** 1 School of Medicine and Public Health, Faculty of Health and Medicine, University of Newcastle, Callaghan, NSW 2308 Australia; 2 Hunter New England Population Health, Hunter New England Local Health District, Locked Bag 10, Wallsend, NSW 2287 Australia; 3 Department of Family Practice, Faculty of Medicine, University of British Colombia, Vancouver, Canada; 4 School of Public Health, University of Sydney, Sydney, NSW 2006 Australia; 5 NSW Ministry of Health, St Leonards, NSW Australia

**Keywords:** Public health intervention, Implementation, Scale-up, Dissemination strategies, Population health, Adaptations

## Abstract

**Background:**

To maximise their potential benefits to communities, effective health behaviour interventions need to be implemented, ideally ‘at scale’, and are often adapted as part of this. To inform future implementation and scale-up efforts, this study broadly sought to understand (i) how often health behaviour interventions are implemented in communities, (ii) the adaptations that occur; (iii) how frequency it occurred ‘at scale’; and (iv) factors associated with ‘scale-up’.

**Methods:**

A cross-sectional survey was conducted of corresponding authors of trials (randomised or non-randomised) assessing the effects of preventive health behaviour interventions. Included studies of relevant Cochrane reviews served as a sampling frame. Participants were asked to report on the implementation and scale-up (defined as investment in large scale delivery by a (non)government organisation) of their intervention in the community following trial completion, adaptations made, and any research dissemination strategies employed. Information was extracted from published reports of the trial including assessments of effectiveness and risk of bias.

**Results:**

Authors of 104 trials completed the survey. Almost half of the interventions were implemented following trial completion (taking on average 19 months), and 54% of those were adapted prior to doing so. The most common adaptations were adding intervention components, and adapting the intervention to fit within the local service setting. Scale-up occurred in 33% of all interventions. There were no significant associations between research trial characteristics such as intervention effectiveness, risk of bias, setting, involvement of end-user, and incidence of scale-up. However the number of research dissemination strategies was positively associated to the odds of an intervention being scaled-up (OR = 1.50; 95% CI: 1.19, 1.88; *p* < 0.001).

**Conclusions:**

Adaptation of implemented trials is often undertaken. Most health behaviour interventions are not implemented or scaled-up following trial completion. The use of a greater number of dissemination strategies may increase the likelihood of scaled up.

**Supplementary Information:**

The online version contains supplementary material available at 10.1186/s12961-023-01027-0.

## Background

Health behaviour interventions, such as those which promote behavioural nutrition, increased physical activity, safe sexual practices, and help or prevent individuals from using tobacco, alcohol or other substances have the opportunity to improve individual health behaviours and prevent related mortality and morbidity [1]. However, interventions must be successfully implemented in real world contexts and ‘scaled-up’ to achieve population health benefits. Scale-up is defined by the World Health Organisation (WHO) as “deliberate efforts to increase the impact of health service innovations successfully tested in pilot or experimental projects to benefit more people and to foster policy and program development on a lasting basis.” [2].

Many effective health behaviour interventions are never delivered in the real world [3–5], and few are delivered at a scale that may have the capacity to achieve population wide reductions in related health risk [4, 5]. Implementation and scale-up frameworks identify a range of factors thought to be important for successful scale-up [6–10]. These include the characteristics of the intervention, such as the scientific quality (or risk of bias) of its evaluation, evidence of its effectiveness, and intervention flexibility [6, 10]. Efforts to scale-up an intervention may also be facilitated when it is supported and informed by a program of formative evaluation, efficacy and effectiveness trials, dissemination research and costing prior to scale-up [10–13] however this is seldom available [14].

Factors related to the characteristics of the setting where an intervention is to be delivered [7], have also been suggested to facilitate successful scale-up [6, 15, 16]. For example, settings with more formalised leadership and infrastructure (e.g. training, delivery systems, technical resources like those found in schools) may aid intervention implementation and scale-up [6, 17], The involvement of end-users across the phases of research is also recommended [18–20]. This improves the compatibility of the intervention to the context or setting where it is to be implemented, and the collection and reporting of information relevant to end-user decision making [21].

Strategies to disseminate the findings of intervention research may improve the likelihood that such interventions are discovered, and implemented, including at scale. International surveys report researchers often use seminars or workshops, face-to-face meetings, media interviews and targeted mailings to disseminate the findings of their research [21, 22]. While research shows that comprehensive dissemination strategies directed at patients can improve the use of evidence to support patient health related behaviours [23], There is limited research examining the association or impact of such intervention trial dissemination strategies on the likelihood of subsequent implementation and scale-up of prevention interventions [22, 24].

In this context, the broad objective of this study was to quantify the frequency of implementation and scale-up of health behaviour interventions and the extent to which factors suggested to facilitate implementation and scale-up are associated with it [14]. Therefore, the objectives of our study are:

A) To describe how often tested public health interventions are reportedly implemented in practice;
B) To describe adaptations to interventions that are thought to facilitate implementation;
C) To describe the frequency to which selected interventions are scaled-up; and
D) To examine the association between trial characteristics (*effectiveness, trial quality, settings, involvement of end-users, and dissemination strategies*) and scale-up.

## Methods

### Study design

We administered a single cross-sectional survey to trial authors (May 2018-June 2019) from 41 countries who (co)authored published manuscripts reporting the effect of public health primary prevention trials [25]. To identify authors of trials, we searched the Cochrane Database of Systematic Reviews for reviews of public health primary prevention interventions. Cochrane reviews were used as the sampling frame to provide international representation and broader coverage of health research and undertake comprehensive and systematic methods to identify all relevant studies. Cochrane reviews were eligible for inclusion if they: targeted nutrition, physical activity, sexual health, tobacco use, alcohol or other substance use; were set in any organisation; and were published between 2007 and 2017.

From the 42 relevant Cochrane reviews (see Additional file 1 for a full list of Cochrane reviews) we extracted trial and author details of interventions meeting study eligibility criteria. We included and extracted author information from trials that met our eligibility criteria, that is trials with a parallel controlled design (randomised or non-randomised) that: had some or all components of the intervention delivered in a setting (e.g. hospital, school, or workplace setting); examined the effects of a preventive health intervention (i.e. those targeting nutrition, physical activity, sexual health, tobacco use, alcohol or substance use); and was published between 2007 and 2017 (to allow time for implementation and scale-up to occur). Trials which did not meet this criteria were excluded.

### Recruitment and data collection

We invited via email, the corresponding, first, second or senior authors from each eligible trial to participate in the study. Authors were invited to complete a Computer Assisted Telephone Interview (CATI) survey or an online survey using an emailed link. All data were entered using REDCap [26], a web survey hosting service. We sent respondents two telephone and/or email reminders (four and eight weeks after the initial contact) with a link to the survey, to maximise study participation rates [27]. Authors could nominate co-authors to complete the survey on their behalf. After four weeks, if we received no response from corresponding authors, we invited the first, second and/or last author of the trial manuscript (if different from the corresponding author) to participate.

### Data collection and measures

The investigator team developed a study specific survey to assess implementation, adaptations, scale-up, and dissemination strategies used by those who delivered a primary prevention intervention following an iterative process. Survey items were grounded in the Payback [28] and Knowledge-To-Action (KTA) Frameworks [29], and similar studies in the published literature [30–40]. Data extraction was conducted by two review authors (SG and KM). Prior to completing data extraction, the data extraction tool was tested on a sample of studies until data extraction was harmonised.

*Aim 1: describe how often public health intervention are implemented into practice*. To describe how often tested public health interventions are reportedly implemented respondents were asked: “When the original trial finished, was the intervention implemented in other settings or organisations not involved in the original trial?” If respondents answered ‘yes’ they were asked to report; “How long from trial completion did it take for the intervention to be implemented into practice”.

*Aim 2: describe adaptations to interventions that are thought to facilitate implementation*. To describe adaptations to interventions that are thought to facilitate implementation, respondents who reported the intervention they trialled had been implemented in other sites, were asked: “Do you know if the intervention was adapted before being implemented within the < < insert organisation type > > other than those who consented to be involved in the original trial?” ‘Original trial’ in this case referred to any organisation that participated in the original study as a control or intervention site. Respondents who answered ‘yes’ then completed a series of items (see Additional file 2) that assessed adaptations as described by the Adaptome framework.

*Aim 3: describe the frequency to which selected interventions are scaled-up*. To describe the frequency to which selected interventions were scaled-up*,* participants were asked whether they received “Investment in the large scale delivery of the intervention by a government or non-government organisation?” This question captures the core dimensions of scale-up namely, it occurring on a perceived ‘large’ scale, and external investment and ownership [23, 42]. Trials of authors that indicated ‘yes’ to this item were defined as having been scaled up.

*Aim 4: examine the association between trial characteristics and scale-up.* To examine the association between trial characteristics and scale-up, we extracted information from published trial reports regarding the effectiveness of the intervention and the intervention setting, and extracted risk of bias (RoB) assessments published for each trial in the Cochrane review in which they were included. Items were included in the survey of corresponding trial authors to assess the involvement of end-users, and dissemination strategies employed. Specifically, we extracted data that described the effect of the intervention on primary and secondary trial outcomes using the following outcome hierarchy [43]. *P*-values were chosen as a measure of intervention effectiveness as they are a metric used to assess trial ‘effectiveness’ reported in the literature [44]. For trials where the primary outcome was not specified in the manuscript, we determined the outcome measure authors used to calculate sample size was the primary outcome. If sample size was not calculated and reported, we adopted the outcome described or inferred from the trial aim as the primary outcome. We selected adjusted *p*-values (if reported) over unadjusted *p*-values. We considered an intervention ‘effective’ if the trial reported a significant effect (*p* < 0.05), in the hypothesised direction, on a primary trial outcome. We considered analyses on secondary outcomes as hypothesis generating [45]. Trials were ‘potentially beneficial’ if change in the primary trial outcome was not significant, but change in one or more secondary outcomes were significant [45]. In instances where primary and sub-group analyses were reported, we included data only from the primary analysis.

We extracted RoB data for each trial from the Cochrane review where the trial was sourced (see Additional file 1 for a list of the Cochrane reviews). Cochrane RoB tools requires reviewers to assign high, unclear, or low for each domain of the tool. We categorised a trials overall RoB based on the most frequently applied classification across the domains. That is, trials in which RoB domains were most frequently categories as ‘high’ RoB were categorised as such. Where two or more RoB assessments were reported with the same frequency, the overall classification given to the trial was based on the higher RoB.

We extracted information about the setting in which the intervention was delivered (or where most intervention components were delivered) and classified these as education (schools and childcare settings), community, medical/healthcare, worksites, or other settings.

End-user involvement in the research was assessed by asking participating authors “To what extent were end-users involved in designing, conducting and evaluating the trial?” Respondent answers were scored as: 0 = ‘not at all’, 1 = ‘a little’, and 2 = ‘substantially’. Participants were asked if any of the strategies listed in Box 1 were used to disseminate findings from their trial [28, 30–40]. We counted the number of dissemination strategies used and used the total in our analyses. This made the variable linear, treating all dissemination strategies as having an equal effect.

### Box 1. List of dissemination strategies participants (i.e. trial authors) were asked about at trial completion

**Table:** 

• Plain language or lay summary
• Targeted presentations to end-users
• Knowledge broker used to communicate findings to end-users
• Education workshops conducted with end-users
• Education materials on how to use the study findings
• Media releases
• Results posted on institutional or study website
• Results posted on social media platforms (e.g. Facebook, Twitter, blogs)
• Publication of results in peer reviewed journals
• Research reports
• Presented at academic conferences, workshops or forums

### Analysis

Data were collected and managed using REDCap electronic data capture tools, hosted at Hunter Medical Research Institute [26]. All analyses were undertaken with SAS v 9.3 [46]. We present descriptive statistics as numbers and percentages for categorical variables and means (standard deviation; SD) or median (quartile 1, quartile 3) for continuous variables, depending on distribution of the data. We calculated time to implementation in months using data provided in the open-ended question, often reported in months or years. Where a range of values was provided, we used the mid-point value for our analyses.

We used logistic regression to examine associations among trial characteristics and scale-up. The association of all variables with the scale-up was assessed in a (univariate) logistic regression model. All variables were then entered all at once into one multivariable regression model; this allowed the model to adjust for each independent variables estimated association with the scale-up. We report unadjusted and adjusted odds ratios and 95% confidence intervals (CIs) for each trial characteristic. We report *p*-values from the multivariable model.

## Results

**Fig. 1:**
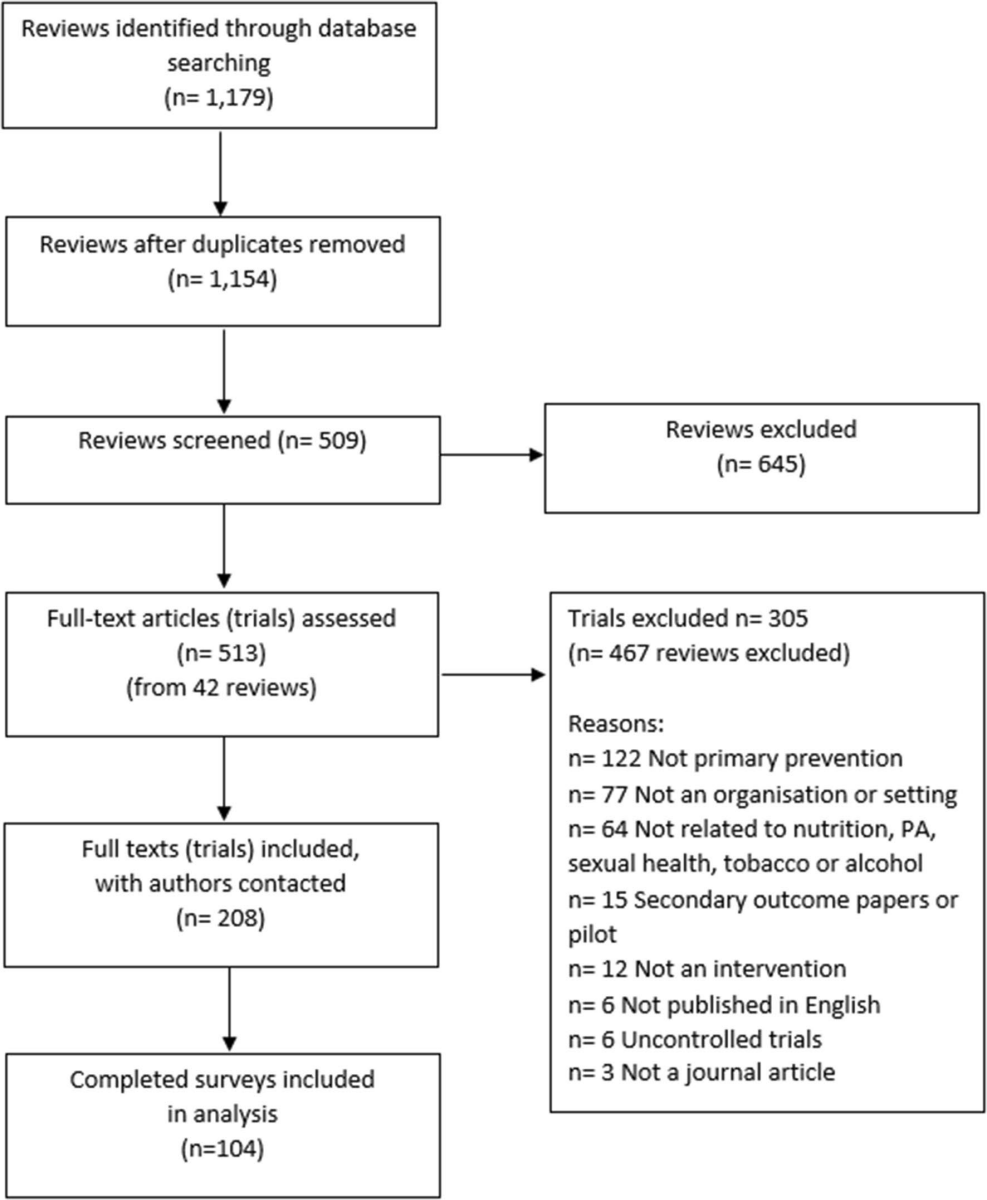
Illustration of the flow and final selection of reviews and trials included in our study

Searching the Cochrane database for eligible review resulted in 1154 review titles which needed screening, 509 of which were full text screened. Of these, only 42 reviews were identified for inclusion, resulting in a total of 208 individual trial included in the study (Fig. 1).

Authors from 208 trials were invited to participate in the study, and we received completed surveys for 104 trials (*n* = 104 authors, 50% completion rate). Most trials focussed on physical activity and/or nutrition (55%) and were conducted in the education setting (67%) in North America (34%). A description of the trials associated with authors who completed the survey is presented in Additional file 3: Table S1.

*How often tested public health interventions are reportedly implemented in practice* Following trial completion, 49% (*n* = 49/99, 5 missing) of interventions were subsequently implemented by an organisation not involved in the original trial (Table 1). Average time to implement the intervention by another organisation following trial completion was 19 months (median = 12; range 0–72 months; *n* = 42, 7 missing).

**Table 1:** Author self-reported adaptations to the intervention or implementation strategies reported at scale-up

Item	Item responses	*n* (%)
When the original trial finished, was the intervention implemented in other settings or organisations not involved in the original trial? (*n* = 99)	No	28 (28%)
	Yes	49 (49%)
	Don’t know	22 (22%)
Do you know if the intervention was adapted before being implemented within the organisations other than those who consented to be involved in the original trial? (*n* = 46)^a^	No, it continued to be implemented exactly as it was developed	15 (33%)
	Yes, it was adapted	25 (54%)
	Don’t know	6 (13%)
If it was adapted please select all that apply (*n* = 25)^b^	It was adapted to fit within the local service setting (i.e. to fit with available resources and funding)	12 (48%)
	It was adapted to fit with the target audience (i.e. tailored to the characteristics of the target population)	8 (32%)
	The mode of delivery was adapted (i.e. dose, length, timing)	9 (36%)
	It was adapted to align with the culture of the setting	5 (20%)
	Core components of the intervention were adapted based on results of testing and research	3 (12%)
	Intervention components or content were added	12 (48%)
	Intervention content or components were removed	6 (24%)
Do you think that the adaptations made were likely to have impacted on the effectiveness of the intervention? (*n* = 25)^c^	Yes, I think it would have likely increased the effectiveness of the intervention	8 (32%)
	Yes, I think it would have likely diluted the effectiveness of the intervention	8 (32%)
	No, I don’t think it would have had an impact on the effectiveness of the intervention	3 (12%)
	Unsure	6 (24%)

^a^These questions were only asked of individuals who responded “yes” (*n* = 49) to the previous question, “When the original trial finished, was the intervention implemented in other organisations not involved in the original trial?”. There was missing data (no response) for 3 participants

^b^The same 25 respondents (who indicated “yes, it was adapted” in the second question) were asked this questions. Response options were a yes/no to each type of adaptation so numbers are presented for ‘yes’ responses only

^c^The same 25 respondents (who indicated “yes, it was adapted” in the second questions)

*Adaptations to interventions that are thought to facilitate implementation* Fifty-four per cent of implemented trials (*n* = 25/46, 3 missing, Table 1) adapted their original intervention prior to it being implemented by an organisation not involved in the original trial. The two most frequently reported adaptations were, (1) adding intervention components, and (2) adapting the intervention to facilitate fit within the local service setting (48%; Table 1). Respondents indicated adaptations would positively impact intervention effectiveness (increase effectiveness; 32%) or diminish effectiveness (dilute effectiveness, 32%).

*The frequency of which selected interventions are scaled-up* One hundred authors responded to the question about scale-up (4 missing). Thirty-three respondents (i.e. 33%) indicated that their intervention was scaled-up—that is, there was external investment in the large scale delivery of the intervention by a government or non-government organisation.

*The association between trial characteristics (effectiveness, trial quality, settings, involvement of end-users, and dissemination strategies) and scale-up* Results from the unadjusted logistic regression indicate that the number of dissemination strategies increased the odds of scale-up by 1.48 with each additional strategy used. After adjusting for all other independent variables, the number of dissemination strategies remained significantly associated with scale-up (Table 2), with the estimate increasing to 1.50 for each additional dissemination strategy used. Scale-up was not significantly associated with other trial characteristics.

**Table 2:** Results of the logistic regression examining trial characteristics associations to scale-up (*n* = 49)

Trial characteristics	Number of interventions *n* = 49 (%)	Unadjusted		Adjusted model^c^	
		Odds ratio (95% CI)	*p*-value	Odds ratio (95% CI)	*p*-value
Intervention effectiveness					
No effect	5 (45%)	Ref.	0.50	Ref.	0.69
Potentially effective only^a^	8 (38%)	0.74 (0.17, 3.24)		0.72 (0.14, 3.81)	
Effective^b^	20 (29%)	0.50 (0.14, 1.83)		0.55 (0.12, 2.45)	
Risk of bias					
Unclear + High risk	22 (34%)	Ref.	0.70	Ref.	0.39
Low risk	11 (31%)	0.84 (0.35, 2.02)		0.64 (0.23, 1.78)	
Settings					
Community + worksite	6 (27%)	Ref.	0.81	Ref.	0.52
Education	23 (34%)	1.39 (0.48, 4.04)		1.13 (0.33, 3.83)	
Medical + other	4 (36%)	1.52 (0.32, 7.15)		2.54 (0.46, 14.20)	
Involvement of end-users (*n* = 48)^d^					
Not at all + a little	13 (27%)	Ref.	0.23	Ref.	0.83
Substantially	20 (38%)	1.68 (0.72, 3.92)		1.11 (0.43, 2.89)	
Dissemination strategies mean number (SD)	7.12 (2.04)	1.48 (1.19, 1.83)	** < 0.001**	1.50 (1.19, 1.88)	** < 0.001**

^a^Trials were deemed potentially beneficial if the effect of the intervention on secondary outcomes was significant (*p* < 0.05), but the effect on the primary outcome was not significant

^b^Trials were deemed effective if the effect of the intervention on the primary outcome was significant (*p* < 0.05)

^c^Model adjusted for intervention effective, risk of bias, setting, involvement of end-user, and dissemination strategies

^d^Missing data for one respondent who preferred not to answer this question

Boldface indicates statistical significance

## Discussion

We extend a relatively sparse evidence base by quantitatively characterising implementation and scale-up of public health interventions, and by identifying factors associated with scale-up. We found that implementation and scale-up reportedly occurred in less than half of the trials included in this study, that the majority of interventions that were implemented in practice had been adapted, and that the number of dissemination strategies, but not the effectiveness of the intervention or study quality (as assessed via risk of bias) was associated with scale-up. Our contribution is an important one, given the substantial investment by granting agencies and governments worldwide in “one-off” pilot studies that do not yield community health improvements [47, 48].

We found that one third of published interventions in this study were scaled-up. This is a greater proportion than that previously estimated by Reis et al. that suggested 3% of physical activity interventions reported in studies were scaled-up [49]. We consider a few possible reasons for the greater likelihood of scale-up reported in our study. First, the sampling frame in our study were trials included in a Cochrane review, and were typically RCTs. Reis included a broader range of trial designs. Cochrane is a trusted and commonly used source of public health evidence [50], something made especially clear during the COVID-19 epidemic where their review evidence was sought by the World Health Organization, National Institute for Health Research and the Brazilian Ministry of Health [51]. Given this, trials included in such reviews may be more likely to be identified and their interventions adopted by health agencies. Second, there is no clear, threshold for determining when scale-up has occured [52]. It may be the moment an intervention is institutionalised at scale by being replicated in a different geographical area [49], or perhaps when it has reached large scale investment [49, 53, 54]. Therefore, perceptions of what constituted scale-up may have differed among respondents between ours and the Reis study. Studies may have used different criteria, tools and approaches to ‘count’ scale-up. For example, data that comprised the 3% of studies scaled-up was specific to physical activity interventions that had published outcomes of the effects of scale-up [49]. We included all public health related studies (only 21% were related to physical activity) and authors self-reported if their intervention was subsequently scaled-up (i.e. no published outcomes were necessary). When compared to a more recent review examining scaled-up nutrition settings-based interventions [5], the proportion of included studies that were scaled-up remains low when compared to other systematic reviews on the same subject (i.e. 10 included scaled-up nutrition studies [5] compared to 69 nutrition studies based in school settings in recent systematic reviews [55]). Nonetheless, the reported prevalence of scale-up in this study is encouraging, and suggests higher rates of this form of translation.

Adaptation for implementation and scale-up is often reported [4, 5, 41, 56]. In this study, adaptations to both function (‘core components’; actions considered essential to drive outcomes) [41] and form (e.g. mode of delivery; delivery preferences or actions considered not essential to outcomes) [57] were common. In public health research to describe core components of an intervention is rare [58], likely due to the absence of large trials that differentiate the specific contribution of intervention components to outcomes. The nature of adaptations to interventions or implementation strategies are also seldom reported, limiting our ability to compare across studies [59]. We recommend that consistent reporting of adaptations at scale-up of perceived core versus non-core components become standard practice. New literature is developing guidelines and frameworks to assist with this issue [60, 61].

We found that a greater number of dissemination strategies was associated with scale-up. Adherence to dissemination guidance such as the Interactive Systems Framework for Dissemination and Implementation [62] may further enhance the impact of investments in dissemination through ensuring the timing, source, message, and channel used in the dissemination strategies are appropriate to decision makers [63]. Future research investigating the effectiveness of specific dissemination strategies, individually and in combination, may help to improve the efficiency and impact of these approaches, with a recent scoping review highlighting there is a significant gap in the literature evaluating the impact of different strategies [22].

Interestingly, we found a lack of association between intervention effectiveness and scale-up when assessed against the significance of the primary trial outcome. This is concerning given that scarce preventive care resources comprise a miniscule proportion of the health care budget (1.9%, 5.8%, 3.0% and 5.2% in Australia, Canada, USA and UK, in 2017 respectively) [64]. The intervention may have reported beneficial effects on other outcomes considered important to end-users investing in their implementation and scale-up. Previous research has identified that a range of factors beyond effectiveness influence scale-up decisions including individual values, politics and finance [7]. Government officials report taking a ‘package proposal’ to politicians to scale-up health programs, and consider perceived end-user or community acceptability, feasibility and cost as well as effectiveness when making decisions [7]. Greater collection and reporting of such evidence may assist in facilitating program scale-up. Surprisingly, reported involvement of end-users was not associated with scale-up. Stakeholder engagement is thought to be essential to ensure the relevance of an intervention and its implementation success [65] and ‘beginning with the end in mind’ [66] seems a logical approach to ensure the research is warranted, has real-world applicability and the possibility that it will be sustained, in future. Greater exploration of this finding is warranted.

We acknowledge a number of limitations need to be considered when interpreting the findings of this study. First, scale-up is a long-term process and more recent trials (published up until 2017) included in this study may not have had sufficient time for this to occur. However, the range in the average time to implementation identified in this study (0–72 months) suggests instances of implementation that may occur, were likely to have been captured in this study. Nonetheless, more comprehensive studies that map the time to implementation and scale-up of public health interventions would help inform periods of latency for these outcomes and the appropriateness of data collection efforts to capture them. Second, we used Cochrane systematic reviews as a pragmatic approach to efficiently identify trials included in our study. However, such reviews often included randomised trial only and may include other criteria such as minimum periods of follow-up, or objective measures of outcome. As such, the findings of the review may not generalise to interventions and trials that may not meet such thresholds. A systematic review of primary studies with more open inclusion criteria would enable a more comprehensive and representative assessment of the objectives of this study. Finally, the impacts of dissemination strategies was assessed using a score based measure by summing the number of strategies employed. Analysis of the association of the individual dissemination strategies may have provided more informative information for the design of more potent approaches to dissemination. Future research should undertake this work.

## Conclusions

It is essential to scale-up effective public health and health promotion interventions to maintain and preserve health at the population level. This study reports that both implementation and scale-up occurred in half of the interventions included from the sample, and that adaptation to these are common in the implementation and scale-up process. The frequency of adaptations highlight the potential that the effects of interventions may also be modified, and the importance of strategies to mitigate the risk of adaptations resulting in a ‘voltage drop’ [67]. While the study supports the use of comprehensive strategies to disseminate the findings of research trials to facilitate research translation, end-users must ensure that interventions selected for scale-up are indeed effective in order to achieve community health improvements.

## Supplementary Information

**Additional file 1.** List of included cochrane reviews. To serve as a sampling frame, the Cochrane Database of Systematic Reviews was searched for reviews of primary prevention health interventions. Reviews were restricted to those published between 2007 and 2017. The survey included 42 Cochrane reviews of preventive health interventions.

**Additional file 2.** Adaptation questions. To describe adaptations to interventions that are thought to facilitate implementation, respondents who reported the intervention they trialled had been implemented in other sites, were asked: “Do you know if the intervention was adapted before being implemented within the <  < insert organisation type >  > other than those who consented to be involved in the original trial?” (response options: yes, no, do not know). Respondents who responded ‘yes’ then completed a series of items (see Additional file 2) that assessed adaptations to the intervention, service setting, target audience, mode of delivery, cultural or core component adaptations as described by the Adaptome framework.

**Additional file 3.** Descriptive statistics of the 104 trials included in the study. Table S1 outlines the trial characteristics of responders.

## Data Availability

The datasets used and/or analysed during the current study are available from the corresponding author on reasonable request.

## Abbreviations

CATI: Computer assisted telephone interview
KTA: Knowledge to action
WHO: World Health Organization
RoB: Risk of bias
CI: Confidence interval
