# Supplementary material for: From trials to communities: implementation and scale-up of health behaviour interventions
Source: Health Res Policy Syst. 2023 Jul 31;21:79. doi: 10.1186/s12961-023-01027-0 (PMC10388470; doi:10.1186/s12961-023-01027-0)
Supplement: Supplementary file 1 — Additional file 1. List of included cochrane reviews. To serve as a sampling frame, the Cochrane Database of Systematic Reviews was searched for reviews of primary prevention health interventions. Reviews were restricted to those published between 2007 and 2017. The survey included 42 Cochrane reviews of preventive health interventions. [file 12961_2023_1027_MOESM1_ESM.docx]

**Additional File 1**

**List of included Cochrane reviews**

1. Adler AJ, Taylor F, Martin N, Gottlieb S, Taylor RS, Ebrahim S. Reduced dietary salt for the prevention of cardiovascular disease. *Cochrane Database Syst Rev.* 2014(12).

2. Al‐Khudairy L, Loveman E, Colquitt JL, et al. Diet, physical activity and behavioural interventions for the treatment of overweight or obese adolescents aged 12 to 17 years. *Cochrane Database Syst Rev.* 2017(6). doi:10.1002/14651858.CD012691.

3. Anderson LM, Adeney KL, Shinn C, Safranek S, Buckner-Brown J, Krause LK. Community coalition-driven interventions to reduce health disparities among racial and ethnic minority populations. *Cochrane Database Syst Rev.* 2015(6). doi:10.1002/14651858.CD009905.pub2.

4. Baker PR, Francis DP, Soares J, Weightman AL, Foster C. Community wide interventions for increasing physical activity. *Cochrane Database Syst Rev.* 2015(1). doi:10.1002/14651858.CD008366.pub3.

5. Baldwin C, Kimber KL, Gibbs M, Weekes CE. Supportive interventions for enhancing dietary intake in malnourished or nutritionally at‐risk adults. *Cochrane Database Syst Rev.* 2016(12).

6. Baxi R, Sharma M, Roseby R, et al. Family and carer smoking control programmes for reducing children's exposure to environmental tobacco smoke. *Cochrane Database Syst Rev.* 2014(3). doi:10.1002/14651858.CD001746.pub3.

7. Brunner E, Rees K, Ward K, Burke M, Thorogood M. Dietary advice for reducing cardiovascular risk. *Cochrane Database Syst Rev.* 2007(4).

8. Carson KV, Brinn MP, Labiszewski NA, Esterman AJ, Chang AB, Smith BJ. Community interventions for preventing smoking in young people. *Cochrane Database Syst Rev*. 2011(7). doi:10.1002/14651858.CD001291.pub2.

9. Carson‐Chahhoud KV, Ameer F, Sayehmiri K, et al. Mass media interventions for preventing smoking in young people. *Cochrane Database Syst Rev.* 2017(6). doi:10.1002/14651858.CD001006.pub3.

10. Dobbins M, Husson H, DeCorby K, LaRocca RL. School-based physical activity programs for promoting physical activity and fitness in children and adolescents aged 6 to 18. *Cochrane Database Syst Rev.* 2013(2). doi:10.1002/14651858.CD007651.pub2.

11. Dudley L, Garner P. Strategies for integrating primary health services in low- and middle-income countries at the point of delivery. *Cochrane Database Syst Rev.* 2011(7). doi:10.1002/14651858.CD003318.pub3.

12. Faggiano F, Minozzi S, Versino E, Buscemi D. Universal school-based prevention for illicit drug use. *Cochrane Database Syst Rev.* 2014(12). doi:10.1002/14651858.CD003020.pub3.

13. Foxcroft DR, Tsertsvadze A. Universal family-based prevention programs for alcohol misuse in young people. *Cochrane Database Syst Rev.* 2011(9). doi:10.1002/14651858.CD009308.

14. Foxcroft DR, Tsertsvadze A. Universal multi-component prevention programs for alcohol misuse in young people. *Cochrane Database Syst Rev.* 2011(9). doi:10.1002/14651858.CD009307.

15. Foxcroft DR, Tsertsvadze A. Universal school-based prevention programs for alcohol misuse in young people. *Cochrane Database Syst Rev.* 2011(5). doi:10.1002/14651858.CD009113.

16. Frazer K, McHugh J, Callinan JE, Kelleher C. Impact of institutional smoking bans on reducing harms and secondhand smoke exposure. *Cochrane Database Syst Rev.* 2016(5). doi:10.1002/14651858.CD011856.pub2.

17. Freak-Poli RL, Cumpston M, Peeters A, Clemes SA. Workplace pedometer interventions for increasing physical activity. *Cochrane Database Syst Rev.* 2013(4). doi:10.1002/14651858.CD009209.pub2.

18. Hayes SL, Mann MK, Morgan FM, Kelly MJ, Weightman AL. Collaboration between local health and local government agencies for health improvement. *Cochrane Database Syst Rev.* 2012(10). doi:10.1002/14651858.CD007825.pub6.

19. Hefler M, Liberato SC, Thomas DP. Incentives for preventing smoking in children and adolescents. *Cochrane Database Syst Rev.* 2017(6). doi:10.1002/14651858.CD008645.pub3.

20. Hollands GJ, Shemilt I, Marteau TM, et al. Portion, package or tableware size for changing selection and consumption of food, alcohol and tobacco. *Cochrane Database Syst Rev.* 2015(9).

21. Hosking J, Macmillan A, Connor J, Bullen C, Ameratunga S. Organisational travel plans for improving health. *Cochrane Database Syst Rev.* 2010(3). doi:10.1002/14651858.CD005575.pub3.

22. Langford R, Bonell CP, Jones HE, et al. The WHO Health Promoting School framework for improving the health and well-being of students and their academic achievement. *Cochrane Database Syst Rev.* 2014(4). doi:10.1002/14651858.CD008958.pub2.

23. Lopez LM, Bernholc A, Chen M, Tolley EE. School-based interventions for improving contraceptive use in adolescents. *Cochrane Database Syst Rev.* 2016(6). doi:10.1002/14651858.CD012249.

24. Lopez LM, Grey TW, Chen M, Denison J, Stuart G. Behavioral interventions for improving contraceptive use among women living with HIV. *Cochrane Database Syst Rev.* 2016(8). doi:10.1002/14651858.CD010243.pub3.

25. Lopez LM, Grey TW, Chen M, Hiller JE. Strategies for improving postpartum contraceptive use: evidence from non-randomized studies. *Cochrane Database Syst Rev.* 2014(11). doi:10.1002/14651858.CD011298.pub2.

26. Lopez LM, Grey TW, Chen M, Tolley EE, Stockton LL. Theory-based interventions for contraception. *Cochrane Database Syst Rev.* 2016(11). doi:10.1002/14651858.CD007249.pub5.

27. Lopez LM, Otterness C, Chen M, Steiner M, Gallo MF. Behavioral interventions for improving condom use for dual protection. *Cochrane Database Syst Rev.* 2013(10). doi:10.1002/14651858.CD010662.pub2.

28. Lopez LM, Stockton LL, Chen M, Steiner MJ, Gallo MF. Behavioral interventions for improving dual-method contraceptive use. *Cochrane Database Syst Rev.* 2014(3). doi:10.1002/14651858.CD010915.pub2.

29. Martin A, Saunders DH, Shenkin SD, Sproule J. Lifestyle intervention for improving school achievement in overweight or obese children and adolescents. *Cochrane Database Syst Rev.* 2014(3). doi:10.1002/14651858.CD009728.pub2.

30. Mason-Jones AJ, Sinclair D, Mathews C, Kagee A, Hillman A, Lombard C. School-based interventions for preventing HIV, sexually transmitted infections, and pregnancy in adolescents. *Cochrane Database Syst Rev.* 2016(11). doi:10.1002/14651858.CD006417.pub3.

31. Mead E, Brown T, Rees K, et al. Diet, physical activity and behavioural interventions for the treatment of overweight or obese children from the age of 6 to 11 years. *Cochrane Database Syst Rev.* 2017(6). doi:10.1002/14651858.CD012651.

32. Moreno R, Nababan HY, Ota E, et al. Structural and community-level interventions for increasing condom use to prevent the transmission of HIV and other sexually transmitted infections. *Cochrane Database Syst Rev.* 2014(7). doi:10.1002/14651858.CD003363.pub3.

33. Mosdøl A, Lidal IB, Straumann GH, Vist GE. Targeted mass media interventions promoting healthy behaviours to reduce risk of non-communicable diseases in adult, ethnic minorities. *Cochrane Database Syst Rev.* 2017(2). doi:10.1002/14651858.CD011683.pub2.

34. Ojo O, Verbeek JH, Rasanen K, et al. Interventions to reduce risky sexual behaviour for preventing HIV infection in workers in occupational settings. *Cochrane Database Syst* Rev. 2011(12). doi:10.1002/14651858.CD005274.pub3.

35. Oringanje C, Meremikwu MM, Eko H, Esu E, Meremikwu A, Ehiri JE. Interventions for preventing unintended pregnancies among adolescents. *Cochrane Database Syst* Rev. 2016(2). doi:10.1002/14651858.CD005215.pub3.

36. Shrestha N, Kukkonen-Harjula KT, Verbeek JH, Ijaz S, Hermans V, Bhaumik S. Workplace interventions for reducing sitting at work. *Cochrane Database Syst Rev.* 2016(3). doi:10.1002/14651858.CD010912.pub3.

37. Thomas RE, Baker PR, Thomas BC, Lorenzetti DL. Family-based programmes for preventing smoking by children and adolescents. *Cochrane Database Syst Rev.* 2015(2). doi:10.1002/14651858.CD004493.pub3.

38. Thomas RE, McLellan J, Perera R. School-based programmes for preventing smoking. *Cochrane Database Syst Rev.* 2013(4). doi:10.1002/14651858.CD001293.pub3.

39. Wariki WM, Ota E, Mori R, Koyanagi A, Hori N, Shibuya K. Behavioral interventions to reduce the transmission of HIV infection among sex workers and their clients in low‐and middle‐income countries. *Cochrane Database Syst Rev.* 2012(2).

40. Waters E, de Silva‐Sanigorski A, Burford BJ, et al. Interventions for preventing obesity in children. *Cochrane Database Syst Rev.* 2011(12). doi:10.1002/14651858.CD001871.pub3.

41. Wolfenden L, Barnes C, Jones J, et al. Strategies to improve the implementation of healthy eating, physical activity and obesity prevention policies, practices or programmes within childcare services. *Cochrane Database Syst Rev*. 2016(10). doi:10.1002/14651858.CD011779.pub2.

42. Wolfenden L, Wyse RJ, Britton BI, et al. Interventions for increasing fruit and vegetable consumption in children aged 5 years and under. *Cochrane Database Syst Rev.* 2012(11). doi:10.1002/14651858.CD008552.pub2.
