# Supplementary material for: From trials to communities: implementation and scale-up of health behaviour interventions
Source: Health Res Policy Syst. 2023 Jul 31;21:79. doi: 10.1186/s12961-023-01027-0 (PMC10388470; doi:10.1186/s12961-023-01027-0)
Supplement: Supplementary file 2 — Additional file 2. Adaptation questions. To describe adaptations to interventions that are thought to facilitate implementation, respondents who reported the intervention they trialled had been implemented in other sites, were asked: “Do you know if the intervention was adapted before being implemented within the < < insert organisation type > > other than those who consented to be involved in the original trial?” (response options: yes, no, do not know). Respondents who responded ‘yes’ then completed a series of items (see Additional file 2) that assessed adaptations to the intervention, service setting, target audience, mode of delivery, cultural or core component adaptations as described by the Adaptome framework. [file 12961_2023_1027_MOESM2_ESM.docx]

**Additional File 2**

**Adaptation questions**

Respondents were asked why the intervention or implementation strategies were adapted : 1) “It was adapted to better fit within the service setting (i.e. changes in who delivered the intervention, changes to better fit with co-occurring interventions in the setting, or due to financing)”; 2) “It was adapted to fit with the target audience (i.e. changes to improve age appropriateness, to be considerate of individual needs, or people with co-morbid conditions)”; 3) “The mode of delivery was adapted (i.e. changes to the technological format, dose, length, timing of the intervention)”; 4) “It was adapted to align with the culture of the setting (i.e. changes considerate of culture such as use of images or language)”; 5) “Core components of the intervention were adapted using research identifying casual mechanisms”; 6) “Intervention components or content were added”, and; 7) “Intervention content or components were removed”.
