# Supplementary material for: From trials to communities: implementation and scale-up of health behaviour interventions
Source: Health Res Policy Syst. 2023 Jul 31;21:79. doi: 10.1186/s12961-023-01027-0 (PMC10388470; doi:10.1186/s12961-023-01027-0)
Supplement: Supplementary file 3 — Additional file 3. Descriptive statistics of the 104 trials included in the study. Table S1 outlines the trial characteristics of responders. [file 12961_2023_1027_MOESM3_ESM.docx]

**Additional File 3**

**Table 1.** Descriptive statistics of the 104 trials included in the study

| **Characteristics of trial** | **n (%)**  **n = 104** |
| --- | --- |
| Year published range | 2007-2016 |
| Study design |  |
| RCT | 86 (83%) |
| Controlled trial | 18 (17%) |
| Health risk targeted |  |
| Nutrition | 13 (13%) |
| Physical activity | 22 (21%) |
| Physical activity and nutrition | 22 (21%) |
| Sexual health | 15 (14%) |
| Smoking | 12 (12%) |
| Substance use | 15 (14%) |
| Substance use and sexual health | 1 (1%) |
| Substance use and smoking | 4 (4%) |
| Setting |  |
| Community | 15 (14%) |
| Education | 70 (67%) |
| Medical | 9 (9%) |
| Worksites | 7 (7%) |
| Other | 3 (3%) |
| Regions |  |
| Europe | 34 (33%) |
| North America | 35 (34%) |
| Oceania | 18 (17%) |
| Other | 17 (16%) |
| Effective intervention^*^ | 71 (68%) |
| Potentially beneficial intervention^^^ | 22 (21%) |
| Risk of Bias |  |
| High RoB | 30 (29%) |
| Unclear | 37 (36%) |
| Low RoB | 37 (36%) |

^*^ Trials were deemed effective if the effect of the intervention on the primary outcome was significant (p<0.05). ^^^ Trials were deemed potentially beneficial if the effect of the intervention on secondary outcomes was significant (p<0.05), but the effect on the primary outcome was not significant. RCT= randomized controlled trial; RoB = risk of bias.

**Table 2.** Count of countries included studies are located.

| **Characteristics of trial** | **n (%)**  **n = 104** |
| --- | --- |
| Angola | 1 (1%) |
| Australia | 6 (6%) |
| Belgium | 1 (1%) |
| Brazil | 2 (2%) |
| Canada | 6 (6%) |
| China | 1 (1%) |
| Czech Republic | 1 (1%) |
| Denmark | 1 (1%) |
| England | 2 (2%) |
| Finland | 1 (1%) |
| France | 3 (3%) |
| Germany | 1 (1%) |
| Hong Kong | 2 (2%) |
| India | 3 (3%) |
| Iran | 1 (1%) |
| Madagascar | 1 (1%) |
| Mexico | 1 (1%) |
| Netherlands | 4 (4%) |
| Norway | 1 (1%) |
| Pakistan | 1 (1%) |
| Romania | 1 (1%) |
| Scotland | 1 (1%) |
| South Africa | 2 (2%) |
| Spain | 1 (1%) |
| Switzerland | 3 (3%) |
| Tanzania | 1 (1%) |
| UK | 5 (5%) |
| UK/Australia, and Spain | 1 (1%) |
| USA | 48 (46%) |
| Zimbabwe | 1 (1%) |
